# Organic Solid-State Tri-Wavelength Lasing from Holographic Polymer-Dispersed Liquid Crystal and a Distributed Feedback Laser with a Doped Laser Dye and a Semiconducting Polymer Film

**DOI:** 10.3390/ma10050509

**Published:** 2017-05-07

**Authors:** Minghuan Liu, Yonggang Liu, Zenghui Peng, Shaoxin Wang, Qidong Wang, Quanquan Mu, Zhaoliang Cao, Li Xuan

**Affiliations:** 1 State Key Laboratory of Applied Optics, Changchun Institute of Optics, Fine Mechanics and Physics, Chinese Academy of Sciences, Changchun 130033, China; liuminghuan13@mails.ucas.ac.cn (M.L.); liuyonggang@ciomp.ac.cn (Y.L.); peng@ciomp.ac.cn (Z.P.); wangshaoxin@ciomp.ac.cn (S.W.); wangqidong@ciomp.ac.cn (Q.W.); muquanquan@ciomp.ac.cn (Q.M.); caozlok@ciomp.ac.cn (Z.C.); 2 University of Chinese Academy of Sciences, Beijing 100049, China

**Keywords:** holographic polymer-dispersed liquid crystal, distributed feedback, tri-wavelength lasing, tuning, diffraction order

## Abstract

Organic solid-state tri-wavelength lasing was demonstrated from dye-doped holographic polymer-dispersed liquid crystal (HPDLC) distributed feedback (DFB) laser with semiconducting polymer poly[-methoxy-5-(2′-ethyl-hexyloxy)-1,4-phenylene-vinylene] (MEH-PPV) and laser dye [4-(dicyanomethylene)-2-methyl-6-(p-dimethylaminostyryl)-4H-pyran] (DCM) by a one-step holography technique, which centered at 605.5 nm, 611.9 nm, and 671.1 nm. The temperature-dependence tuning range for the tri-wavelength dye-doped HPDLC DFB laser was as high as 8 nm. The lasing emission from the 9th order HPDLC DFB laser with MEH-PPV as active medium was also investigated, which showed excellent s-polarization characterization. The diffraction order is 9th and 8th for the dual-wavelength lasing with DCM as the active medium. The results of this work provide a method for constructing the compact and cost-effective all solid-state smart laser systems, which may find application in scientific and applied research where multi-wavelength radiation is required.

## 1. Introduction

Organic solid-state lasers (OSSLs) have attracted substantial scientific interest since they are compact, cost-efficient, operate at a low threshold, and have wide spectral covering and tunability [1,2,3,4]. Moreover, OSSLs have the potential to achieve electrical injection [5], which makes them smart and promising laser sources for spectroscopy [6], integrated photonics [7], and sensing [8].

OSSLs usually use organic laser dye-doped medium [9] or semiconducting polymer film [10] as active medium. The cavity configuration such as distributed feedback (DFB) [11], distributed Bragg reflector (DBR) [12], and micro-cavity [10] was used to provide positive feedback and lasing mode selection. The DFB configuration provides excellent mode selection and operates with a low threshold among those configurations [2]. The DFB configuration can be fabricated by molding [13] and imprinting [11] process. In addition, the holographic polymer-dispersed liquid crystal (HPDLC) grating can also provide well-defined DFB configuration via polymer-induced phase separation (PIPS) [14]. Moreover, the HPDLC grating show advantages over those molding and imprinting process for it is cost-effectiveness, ease of fabrication, and mass production.

Multi-wavelength OSSLs are useful and promising devices, especially in the field of communications [15] and lab-on-a-chip [16], since multi-wavelength lasing can be achieved with one smart laser. To achieve this goal, Diao et al. reported the dual-wavelength lasing from the semiconducting film and the doped dye using the HPDLC as oscillation cavity [17]. Zhang et al. demonstrated the surface-emitting dual-wavelength laser from a blended gain layer using two semiconducting polymer [18]. However, they just used two kinds of gain medium and cannot provide insights for guidance. The tri-wavelength lasing was not demonstrated for OSSLs from the HPDLC as oscillation cavity until now.

In previous work, we demonstrated that the organic holographic polymer-dispersed liquid crystal and a distributed feedback laser from different diffraction orders, and we believed that the combination of the diffraction order and LC provide a more intriguing feature to the HPDLC DFB laser [19]. In this study, a tri-wavelength lasing from a holographic polymer-dispersed liquid crystal and a distributed feedback laser with dye-doped HPDLC and a semiconducting polymer film as an active medium was demonstrated—and to the best of our knowledge, for the first time. The semiconducting polymer film was spin-coated onto a glass substrate and sandwiched between the glass substrate and the HPDLC grating film. The lasing emission from the 9th diffraction order for the HPDLC DFB laser was investigated. In addition, the laser dye [4-(dicyanomethylene)-2-methyl-6-(p-dimethylaminostyryl)-4H-pyran] (DCM) was doped into the HPDLC grating. The laser dye DCM and semiconducting polymer film were both encapsulated into the device to prevent photo-induced oxidation and degradation. A dual-wavelength lasing was also achieved from a diffraction order different from DCM. The experimental results demonstrated that multi-wavelength lasing occurs when there are multi-cavity modes in the gain spectra. The laser device, which is lightweight, smart, cost-effective, and easy to fabricate, and enjoys tri-wavelength operation and tunability, is promising in the fields of sensing and spectroscopy.

## 2. Materials and Methods

### 2.1. Sample Preparation

The poly[-methoxy-5-(2′-ethyl-hexyloxy)-1,4-phenylene-vinylene] (MEH-PPV) film was used as an active medium layer. The MEH-PPV (Polymer Light Technology) was dissolved in tetrahydrofuran (THF) by weight ratio at 0.6 wt %. The solutions were stirred for 72 h to ensure sufficient dissolution. A drop of MEH-PPV solution was injected on a piece of deionized pre-clean glass substrate for spin-casting. The thickness of the MEH-PPV film was controlled by varying the spin speed and measured by a surface profiler (KLA Tencor P-16+). The thickness was controlled at 75 nm for good lasing operation [20]. All experiments were performed under the same ambient circumstances.

For holographic polymer-dispersed liquid crystal (HPDLC) distributed feedback (DFB) laser fabrication, the HPDLC grating film was recorded onto the MEH-PPV film via holography photo-induced polymerization [14]. The holographic medium mixture for HPDLC mainly contained acrylate monomers (dipentaerythritol hydroxyl pentaacrylate (DPHPA, Aldrich, 29.4 wt %, Shanghai, China) and phthalicdiglycoldiacrylate (PDDA, Eastern Acrylic Chem, 29.4 wt %, Shandong, China)) and nematic liquid crystals (TEB-30A, *n_o_* = 1.522, Δ*n* = 0.170, Silichem, 29.4 wt %). Crosslinking monomer N-vinylpyrrolidone (NVP, Aldrich, 9.8 wt %, Shanghai, China) was also added to dilute the mixture. Rose Bengal (RB, Aldrich, 0.5 wt %, Shanghai, China) and N-phenylglycine (NPG, Aldrich, 1.5 wt %, Shanghai, China) was used as photo-initiator and co-initiator, respectively [19]. The laser dye 4-(dicyanomethylene)-2-methyl-6-(p-dimethylaminostyryl)-4H-pyran (DCM, Aldrich, 0.5 wt %, Shanghai, China) was added in the mixture for dye-doped HPDLC DFB laser fabrication [17]. The mixture, which was stirred with a magnetic bar for 48 h for a homogeneous material system, was injected into an empty glass cell by capillary action in a darkroom. The empty cell was made with two pieces of glass substrate. One had a spin-cast MEH-PPV film, and the other was a pure glass substrate. The thickness of the cell was controlled by spacers at 9 μm, which was thick enough for the doping DCM laser dye to absorb enough excitation energy for lasing oscillation buildup when photo pumping. The refractive index of the polymer-dispersed liquid crystal (PDLC, (the LC with random alignment and the polymer)) was 1.543 at 589.0 nm, which was confirmed by an Abbe refractometer (2WA, Kernco, El Paso, TX, USA). The refractive index of MEH-PPV film was 2.0 at 589.0 nm [21].

### 2.2. HPDLC Grating Fabrication

**Figure 1:**
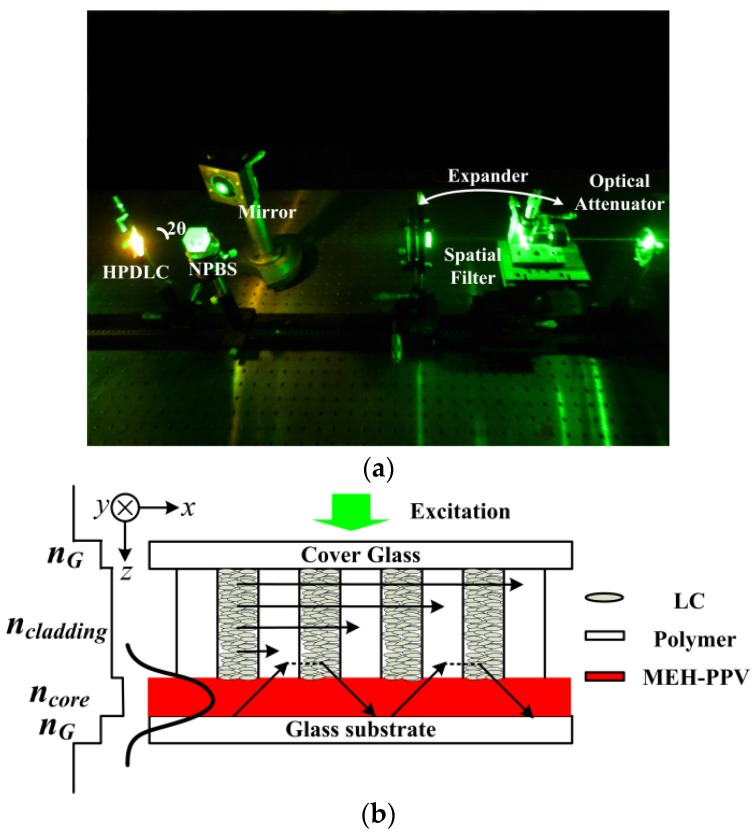

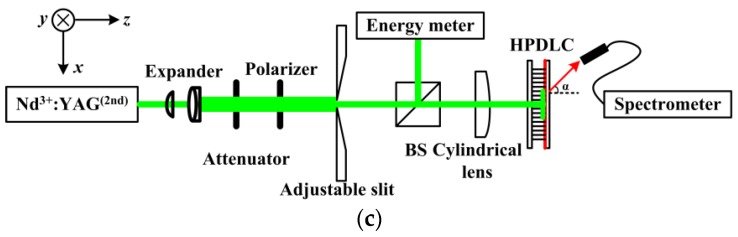
(**a**) Experimental schematic setup for holographic polymer-dispersed liquid crystal (HPDLC) and distributed feedback (DFB) film fabrication. (**b**) Schematic illustration of HPDLC DFB laser device structure and (**c**) an HPDLC DFB laser lasing characterization experimental diagram.

The unslanted transmittance HPDLC grating film was photo-cured by illuminating the cell for three minutes by two s-polarized continuous frequency doubled neodymium-doped yttrium aluminum garnet (Nd^3+^:YAG) laser beams (New Industries Optoelectronics, Changchun, China), as shown in Figure 1a [20]. The recording beam was expanded and filtered to ensure uniform illumination. A variable optical attenuator was inserted to the beam path to regulate the beam intensity. The coherent laser beams pass through the pre-polymer mixture first when fabricating the HPDLC grating as shown in Figure 1a. The energy will be absorbed strongly when the MEH-PPV film faces the coherent laser beams. Therefore, the interference field cannot be built up effectively. The HPDLC film can also protect the MEH-PPV film from photo-induced oxidation and degradation [22]. The period of the HPDLC grating film is determined by

(1) $$\Lambda =\frac{\lambda_{rec}}{2\sin(\theta )},$$

where Λ is the period of the HPDLC grating, *λ_rec_* = 532 nm is the recording laser wavelength in vacuum, and *θ* is half of the intersection angle between two recording beams. The intersection angle can be changed to obtain different grating periods in experiments. Figure 1b depicts the device structure without DCM dye doping. Figure 1b,c is in agreement with each other and they are complementary. The sample is transversely excited in both Figure 1b,c. Figure 1b demonstrates mainly in the lasing oscillation buildup mechanism and Figure 1c demonstrates the collection of the lasing signal. The lasing signal is coupled out via grating coupling as shown in Figure 1c. Details can be found in Section 3.2.

### 2.3. Lasing Characterization

For lasing characterization, the samples were transversely optical excited by a frequency doubled Q-switched Nd^3+^:YAG pulsed laser, which delivered 10 ns pump pulses at a repetition rate of 10 Hz and an excitation wavelength of 532 nm (New Industries Optoelectronics), as shown in Figure 1c. The output lasing signal was collected with a fiber-coupled grating spectrometer, which possessed a resolution limit at 0.23 nm (Sofn Instruments, Beijing, China). The excitation energy was monitored by redirect part of the incident energy into an energy meter via a non-polarized beam splitter (BS) in real time. The pump laser beam was expanded and collimated, and only the central part was selected in order to ensure uniform pumping. Then, the excitation beam was reshaped with a cylinder lens (f = 200 mm) to produce a 3 mm by 1 mm excitation beam at a 0° angle with respect to the normal of the sample. Moreover, a polarizer was used to set the incident polarization. The variable optical attenuator was used to regulate the pump energy to investigate output emission intensity as a function of excitation energy.

## 3. Results and Discussion

### 3.1. Spectroscopic Characterization

**Figure 2:**
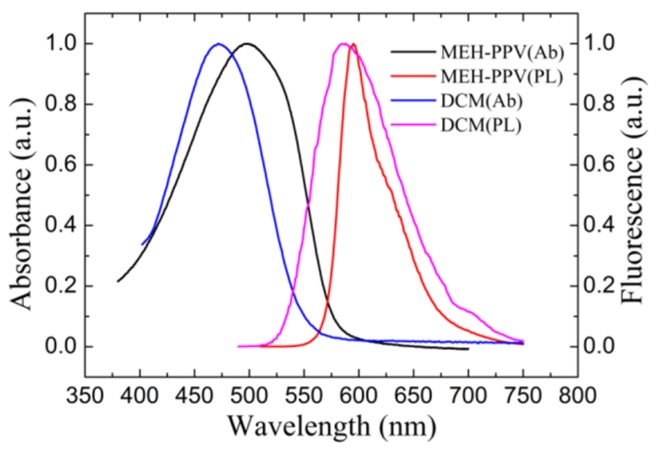
Spectroscopic characterization, e.g., the absorbance and fluorescence spectra of the active medium MEH-PPV and DCM. The fluorescence spectrum was collected with continuous excitation at 500 and 480 nm for MEH-PPV and DCM, respectively.

The pure film spectrum indicates the essential electronic and vibrational structure properties of an organic material. An active medium can be used appropriately by understanding the essential electronic and vibrational structure. Generally, the excitation laser wavelength should match the maximum absorbance wavelength well in order to achieve the population inversion effectively. In addition, the cavity modes should be involved in the gain spectrum of the active medium for lasing emission operations according to laser principles. Moreover, the lasing mode should be optimized to the position where the active medium is a four-level system for low-threshold operation [2,23]. Figure 2 shows the spectroscopic properties of the active medium MEH-PPV film and the laser dye DCM. The absorbance spectra of the spin-coating MEH-PPV film and the laser dye DCM were performed by a UV-3101PC (SHIMADZU, Shanghai, China) UV-VIS-NIR spectrometer. For spectroscopic characterization, the laser dye DCM was doped into DPHPA/NVP (6:1 by weight) by a weight ratio at 0.5% and then injected into a clean glass cell by a capillary action. The photoluminescence (PL) spectra were performed by F-7000FL (Hitachi, Shanghai, China) spectrometer, and the excitation wavelengths were selected at 500 nm and 480 nm to match the peak absorbance of MEH-PPV and DCM, respectively. The broad absorbance spectra show a single peak locating at 500.8 nm and 480 nm in the visible spectral band for MEH-PPV and DCM respectively, where the absorbance is the maximum. The full width at half maximum (FWHM) values of the absorbance spectra are 90 nm and 120 nm for DCM and MEH-PPV, respectively. The absorbance spectra confirm that the absorbance are sufficient for both of the active media when photo-pumping the sample at 532 nm. The peaks of the PL spectra represent an electronic and vibrational structure, the 0-0 and 0-1 emission band. The S_0-0_ represents the transition from the bottom of the first excited singlet state to the bottom of the ground state, which corresponds to a three-level system. While the S_0-1_ represents the transition from the bottom of the first excited singlet state to the first vibration level of the ground state, the molecules will then reach the bottom of the ground state via thermal relaxation, which corresponds to a four-level system. The S_0-0_ and S_0-1_ peaks of the PL spectra are centered at 590 nm and 634 nm for MEH-PPV, and they are 580 nm and 610 nm for DCM, respectively. As a result, the large stokes shift (the spectral range between the absorbance peak and the fluorescence peak) of DCM and MEH-PPV red-shifts their fluorescence emission from absorbance and makes them a good candidate to be used as active medium.

### 3.2. The Mechanism of HPDLC DFB Laser

According to Kogelnik’s coupled wave theory about the DFB laser [24], the lasing wavelength *λ_las_* in vacuum of the HPDLC DFB laser [20] can be expressed as

(2) $$\lambda_{\mathrm{las}}=\frac{2n_{eff}\Lambda }{m},$$

where *n_eff_* is the effective refractive index of the lasing mode, Λ is the HPDLC grating period, and m is the diffraction order. Equation (2) demonstrates that the HPDLC grating period and the diffraction order can be changed so as to select a variable lasing wavelength from the HPDLC DFB laser. For a constant diffraction order, the tunability can be achieved by varying the grating period. The tunable range is the gain spectrum in which the lasing works [25].

The light field will be amplified when it travels through the HPDLC grating/MEH-PPV film/glass substrate waveguide configuration during the excitation process for the HPDLC DFB laser. The refractive index of the MEH-PPV film is higher than the HPDLC grating film and the glass substrate, so the light field is mainly confined in the MEH-PPV film as shown in Figure 1b. In addition, the evanescent light field travels into the HPDLC grating and travels a distance in the HPDLC grating via Goos–Haenchen shift [26]. (The Goos–Haenchen shift occurs in the case of total internal refection. When the total internal reflection happens, the incident light wave from the high refractive index medium at the medium boundary will travel into the low refractive index medium at the depth of one *λ.* There is no power transport into the low refractive index medium. The distance between the entrance and the exit of the incident light wave is about half of one λ, which is the Goos–Haenchen shift.) As a result, the light field is compressed and selected by the HPDLC grating. Then, the evanescent light field travels back into the MEH-PPV film. Thus, the light field will be further amplified by the MEH-PPV film and further compressed by the HPDLC grating. In such a process, the positive feedback builds up and the laser eventually works, as shown in Figure 1b.

**Figure 3:**
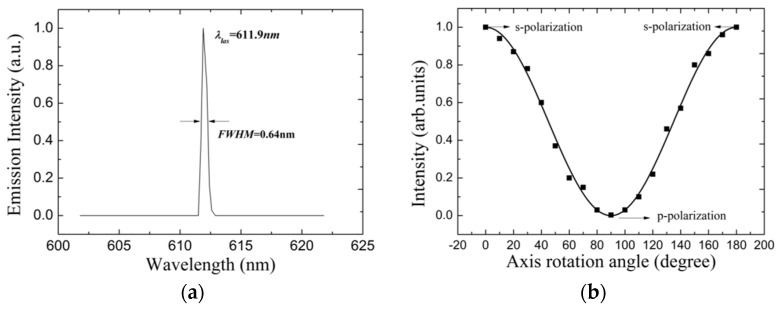
Lasing property of the 9th order HPDLC DFB laser. (**a**) Lasing spectra with the grating period selected at 1.72 μm and (**b**) the emission intensity as a function of the polarizer axis rotation angle.

Figure 3 demonstrates lasing properties of the 9th order HPDLC DFB laser. The grating period was selected at 1.72 μm to achieve the 9th order HPDLC DFB laser. The line width of the fluorescence of MEH-PPV decreases with excitation energy until the lasing threshold. The central wavelength of the lasing emission is 611.9 nm, with a full width at half maximum (FWHM) at 0.64 nm, as shown in Figure 3a. The spectrum is compressed intensely in comparison with the fluorescence spectrum as shown in Figure 2, which confirms the excellent spectral compression and selectivity of the HPDLC grating. The quality factor *Q* (*Q* = λ/Δλ) is as high as 956. The single peak and the quality factor confirm the good lasing and single longitudinal mode operation. The effective refractive index of the 611.9 nm lasing mode is 1.60. A polarizer was placed before the detector system to investigate the polarization of the emission beams. The emission intensity as a function of the polarizer axis rotation angle is shown in Figure 3b. The square points are experimental while the solid line is a cosine fitting of the experimental data. The polarization degree ((*I_‖_ − I*_⊥_)/(*I_‖_ + I*_⊥_) × 100%) is as high as 99%, which confirms the excellent s-polarization property of the emission beams.

**Figure 4:**
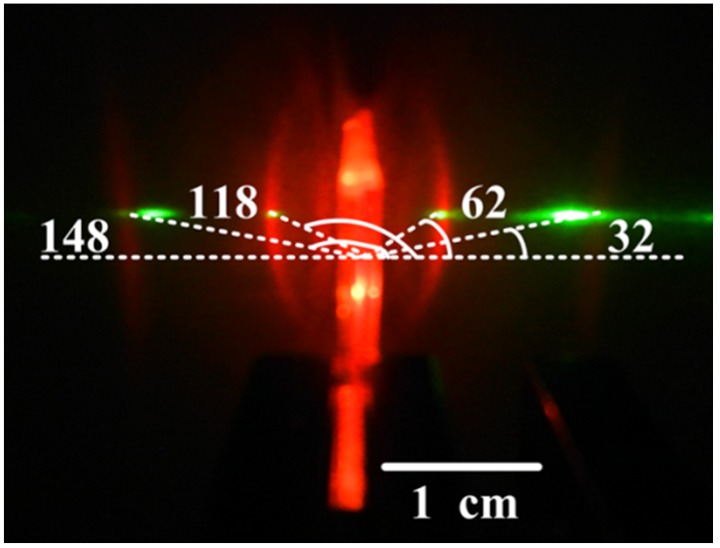
The emission pattern of the 9th order HPDLC DFB laser collected by a digital camera with the excitation energy at 20 μJ/pulse.

For the diffraction order m > 1, the lasing radiation is coupled out of the waveguide by an angle α relative to the HPDLC DFB laser surface normal via grating coupling [27,28,29,30] as shown in Figure 1c. The guided mode travelling in the HPDLC DFB laser [28] can be expressed as

(3) $$k^{2}={\left(nG_{0}-k_{0}\sin\alpha \right)}^{2},$$

where *k* is the wavenumber of the light travelling in the MEH-PPV film, *n* is an integer, *G_0_* is the wavenumber of the HPDLC grating (*G*_0_ = 2πΛ^−1^), and *k_0_* is the wavenumber of the light in vacuum. Figure 4 depicts the emission pattern of the 9th order HPDLC DFB laser. The emission angles are 32, 62, 118, and 148°, which is in good agreement with the theoretical calculations performed with Equation (3).

### 3.3. Tri-Wavelength Lasing from Dye-Doped HPDLC DFB Laser

**Figure 5:**
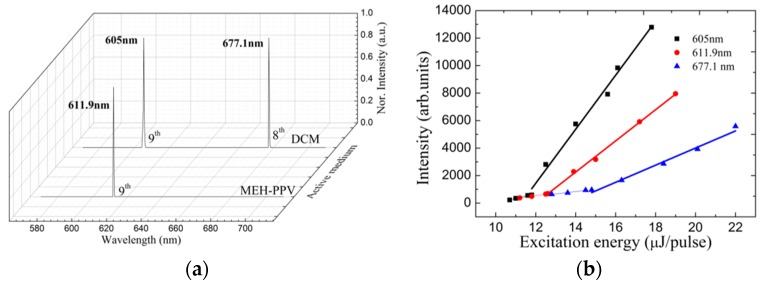
Tri-wavelength lasing property for the dye-doped HPDLC DFB laser at 20 °C. (**a**) Tri-wavelength lasing spectra of the dye-doped holographic polymer-dispersed liquid crystal (HPDLC)-distributed feedback (DFB) laser. The grating period was 1.72 μm for the tri-wavelength lasing operation. (**b**) Dependence of output emission intensity to excitation energy for different wavelength.

The multi-wavelength lasing emission operates when cavity modes exist within the gain spectrum [2,23]. It is confirmed that the DCM has a broad fluorescence spectrum as shown in Figure 2. Moreover, the lasing emission reported for DCM was from 574 to 685 nm with different period HPDLC grating as oscillation cavity [17,31,32]. Therefore, the dual-wavelength lasing emission operates between two adjacent diffraction orders when two cavity modes exist within the gain spectrum of the laser dye DCM according to Equation (2). In previous work, we reported that the lasing emission for MEH-PPV ranged from 590 to 657 nm; as a result, the dual-wavelength lasing cannot operate in this study [19]. The DCM dye-doped HPDLC DFB laser with a grating period selected at 1.72 μm was fabricated to achieve the tri-wavelength lasing emission. Figure 5a shows the lasing spectra of the dye-doped HPDLC DFB laser. Thanks to the distributed feedback mechanism for both active media MEH-PPV and DCM, the lasing emission was collected from the sample edge at one time. The lasing emission centers at 605.0 nm, 611.9 nm, and 677.1 nm as shown in Figure 5a. The good lasing spectra indicate the reliability of the tri-wavelength HPDLC DFB laser. The 605.0 nm and 677.1 nm lasing was delivered from DCM, confirming that the theory is reasonable. The 611.9 nm lasing was delivered from MEH-PPV, which confirmed that the lasing from DCM and MEH-PPV propagate independently. In addition, the diffraction order for the 605 nm, 611.9 nm, and 677.1 nm lasing is 9th, 9th, and 8th, respectively. The effective refractive index of the 605.0 nm, 611.9 nm, and 677.1 nm lasing is 1.58, 1.60, and 1.572, respectively. Figure 5b shows the dependence of output emission intensity to excitation energy of the tri-wavelength laser. The lasing intensity increases gently with excitation energy until the threshold. The lasing threshold for 605.0 nm, 611.9 nm, and 677.1 nm lasing is 11.8 μJ/pulse, 12.6 μJ/pulse, and 14.8 μJ/pulse, respectively. The lasing threshold for 677.1 nm lasing is higher than that of 605 nm lasing because the net-gain decreases intensely at the gain edge even though it operates at a lower diffraction order [30,33,34]. The net-gain for the 611.9 nm lasing is smaller than the 605.0 nm lasing, and the lasing threshold is thus higher.

### 3.4. Tuning Property for the Tri-Wavelength Lasing HPDLC DFB Laser by Elevating Temperature

**Figure 6:**
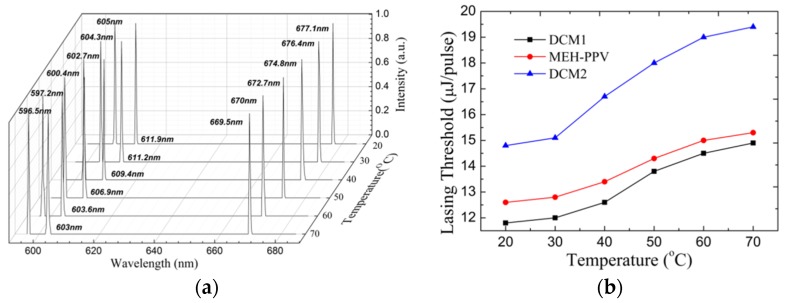
Tuning property for the tri-wavelength lasing HPDLC DFB laser by elevating temperature. (**a**) Dependence of the lasing spectra to operation temperature. (**b**) The working lasing threshold as a function of the operation temperature.

Thanks to the positive nematic LC layer in the HPDLC holographic grating, the lasing tunes by the variation of operation temperature. The molecular configuration transforms from the nematic phase state to the isotropic phase state when temperature changes (N-I transition) [35]. The N-I transition temperature for the LC TEB-30A used in this work is 61.3 °C (DSC Q2000). The sample was in full contact with a hotplate during optical pumping, and the sample temperature was controlled by a thermostat from 20 to 70 °C. Figure 6 illustrates the tuning property for the tri-wavelength lasing HPDLC DFB laser by elevating temperature. Narrow peaks are observed in every lasing wavelength, which indicates that laser action is preserved. The center wavelength shifts to the high energy range with temperature. The reason is that the mean refractive index of LC decreases with temperature, which leads to the decrease in the mode effective refractive index according to Equation (2). The center wavelength for the 605.0 nm lasing changes from 605.0 to 596.5 nm when the temperature increases from 20 to 70 °C, which corresponds to an 8.5 nm blue shift. As for the other lasing from DCM, the center wavelength changes from 677.1 to 669.5 nm when the temperature increases from 20 to 70 °C, which corresponds to a 7.6 nm blue shift. As for the lasing from MEH-PPV, the center wavelength varies from 611.9 to 603.0 nm with the temperature increasing from 20 to 70 °C, which corresponds to an 8.9 nm blue-shift as shown in Figure 6a. In a word, the temperature-dependence tuning range for the tri-wavelength dye-doped HPDLC DFB laser is as high as 8 nm. Figure 6b shows the dependence of a working lasing threshold with an operation temperature. The lasing threshold for the 605 nm lasing increases from 11.8 to 14.9 μJ/pulse when the temperature increases from 20 to 70 °C. As for the other lasing from DCM, the lasing threshold increases from 14.8 to 19.4 μJ/pulse when the temperature increases from 20 to 70 °C. As for the lasing from MEH-PPV, the lasing threshold increases from 12.6 to 15.3 μJ/pulse with the temperature increasing from 20 to 70 °C. The reason for the increase in the lasing threshold is that the coupling decreases with the elevating temperature according to the coupled wave theory [24]. The laser device, which is lightweight, smart, and cost-effective and enjoys one-step holography fabrication, tri-wavelength operation, and temperature-dependent tunability, is promising in the fields of sensing and spectroscopy [6].

## 4. Conclusions

In summary, organic tri-wavelength lasing was achieved from a holographic polymer-dispersed liquid crystal and a distributed feedback laser with a semiconducting polymer poly[-methoxy-5-(2′-ethyl-hexyloxy)-1,4-phenylene-vinylene] (MEH-PPV) and laser dye [4-(dicyanomethylene)-2-methyl-6-(p-dimethylaminostyryl)-4H-pyran] (DCM) by one-step holography, which centered at 605.0 nm (DCM), 611.9 nm (MEH-PPV), and 677.1 nm (DCM). The lasing emission from the 9th order HPDLC DFB laser with MEH-PPV as an active medium was also investigated, which showed excellent s-polarization characterization. The grating period was selected at 1.72 μm to achieve tri-wavelength lasing. The diffraction order is 9th and 8th for the dual-wavelength lasing with DCM as the active medium. The temperature-dependence tuning range for the tri-wavelength dye-doped HPDLC DFB laser was as high as 8 nm. We believe that the results of this work provide a method for constructing the compact and cost-effective solid-state smart laser systems, which may find applications in scientific and applied research where multi-wavelength radiation is required.
